# An *old* Twist in HLA-A: CDR3α Hook up at an *R65-joint*

**DOI:** 10.3389/fimmu.2015.00268

**Published:** 2015-05-27

**Authors:** Joseph S. Murray

**Affiliations:** ^1^Xenolaüs Genetics LLC, Los Angeles, CA, USA

**Keywords:** immunogenetics, TCR, MHC, HLA, alloreactivity, placentation, primate, evolution

## Abstract

T-cell ontogeny optimizes the α/β T-cell receptor (TCR) repertoire for recognition of major histocompatibility complex (MHC) class-I/II genetic polymorphism, and co-evolution of TCR *germline* V-gene segments and the MHC must entail *somatic* diversity generated in the third complimentary determining regions (CDR3α/β); however, it is still not clear how. Herein, a conspicuous structural link between the V-Jα used by several different TCR [all in complex with the same MHC molecule (HLA-A2)], and a conserved MHC motif (a.a., R65-X-X-K-A-X-S-Q72) is described. We model this *R65-joint* in detail, and show that the same TCR’s CDR3α loop maintains its CDR2α loop at a distance of ~4 Å from polymorphic amino acid (a.a.) positions of the α-2 helix in all but one of the analyzed crystal structures. Indeed, the *pitch* of docked TCRs varies as their *twist/tilt/sway* maintains the *R65-joint* and peptide contacts. Thus, the *R65-joint* appears to have poised the HLA-A lineage toward alloreactivity.

## Introduction

The same DNA-recombinase system (*RAG-1/-2*) used in B cells for the generation of variants of the canonical immunoglobulin (Ig) cell-surface receptor is used in T cells to generate a vast diverse repertoire of T-cell receptor (TCR) variants; these variants of the TCR are clonally distributed on T cells, as are sIg on B cells (1). By contrast, within any given individual, the number of possible major histocompatibility complex (MHC) (HLA in human) components of the TCR ligand is limited by two (at most) different alleles of any given HLA heavy-chain gene (1–3). The most enigmatic phenomenon involving TCR and the MHC concerns a very high relative frequency of T cells with exquisite sensitivity to minor changes in the peptide component of pHLA, which nevertheless proliferate against allogeneic pHLA. Because *allo*-HLA is not present in the thymus, and as such the TCR repertoire cannot be selected against different individuals’ HLA molecules, there exists a high precursor frequency of T cells that *cross-react* against *allo*-HLA bearing targets (1–10). Thus, there is a potent biological capacity in the apparent absence of any stimulus, except during gestation. Here, we describe how somatically distinct CDR3α (with one exception) achieves a germline-encoded mean interface of 3.94 ± 0.23 Å between CDR2α and a discreet polymorphic region of HLA-A. Together with bioinformatics evidence, this *R65-joint* indicates that adaptive immunity is constrained by an apparent need for precise alloreactivity (11).

## Results and Discussion

**Figure 1:**
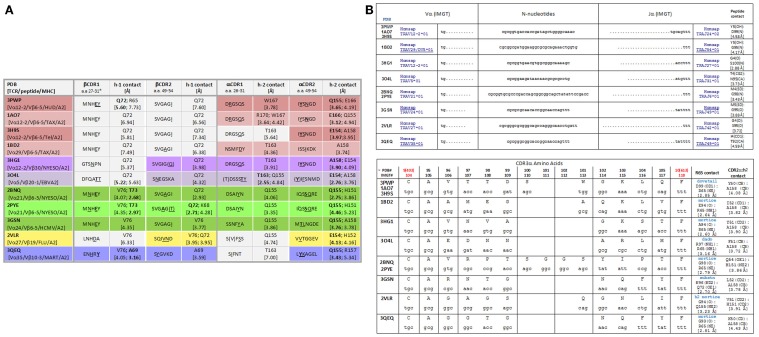
**(A)** CDR1/CDR2 contacts with MHC amino acids among TCR:pHLA-A2 crystallographic structures. The colors indicate the *alpha-dominant* (rose shades), *alpha/beta* (lavender, green, and yellow), and *beta-dominant* (blue) modes of binding. [**(B)**, *top*] nucleotide sequences for all CDR3α of TCR in the indicated PDB files. TCRA were reverse translated then subjected to joint analysis. [**(B)**, *bottom*] CDR3α joints (consensus *IMGT* numbering is shown with PDB numbering as indicated); contacts were measured with *VMD software* (www.ks.uiuc.edu). Characterization of R65 contacts was by analysis of “surf representations” (i.e., space-filled modeling) as shown in figures below. For multiple structures involving the same TCR, contacts in one structure are shown, i.e., for 1AO7 and 2BNQ.

Shown in Figure 1A is our analysis of the CDR1 and CDR2 contacts made by several distinct TCR across different TCR:pHLA structures available in the *Protein data bank* (PDB). All of these structures involve HLA-A*0201 and each has a distinct peptide component. As can be seen from the closest contacts made by the TCR, one can classify these TCR as *alpha-dominant*, *alpha/beta*, and in one case, *beta-dominant*, on the basis of these interactions. Indirectly, this corroborates the role of the CDR3 regions in selective binding of any given TCR for the peptide component (12–16). Theoretically, TCR bearing CDR3 regions that did not disrupt these CDR1/2 interactions with the α-helices of the HLA groove during fetal life would have been repetitively engaged with thymic antigen presenting cells, and such clones would be deleted (1, 4, 9).

### Bioinformatics analysis

**Figure 2:**
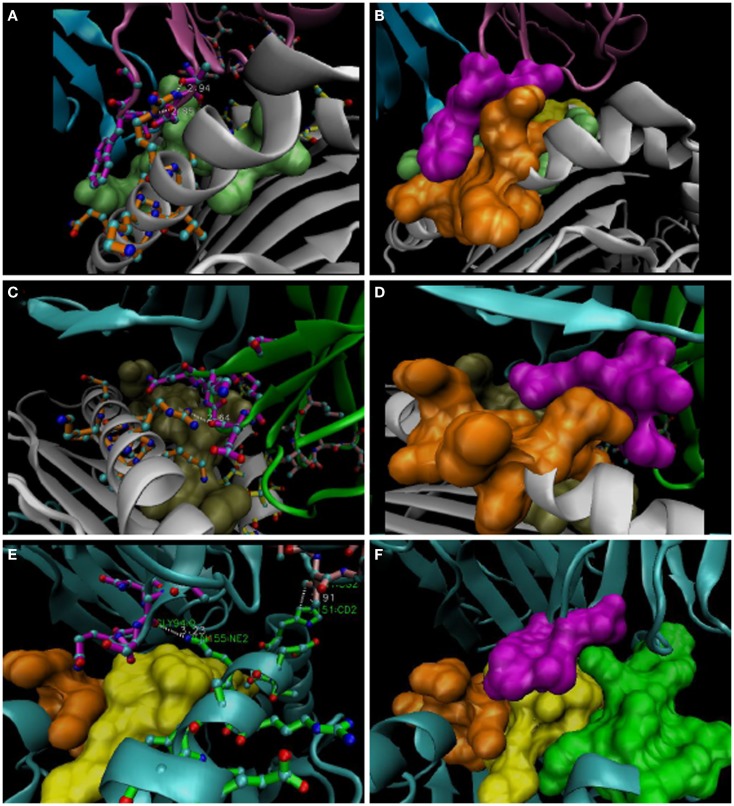
**Representative *R65-Joints* of these TCR:pMHC**. **(A,B)** 1AO7; **(C,D)** 1BD2; **(E,F)** 2VLR. *VMD software* used to isolate structures and make bond measurements; “licorice” representations are shown at *left* and “surf” representations are shown on the *right*. Docking CDR3α a.a. are in magenta (Vα: in magenta, 1AO7; green, 1BD2; cyan, 2VLR); the *R65 motif* is in orange, and the H151-A158 region is in green (bottom panels). Peptides are lime, tan, and yellow for three structures, respectively. Note the W101 *dovetail* of 1AO7 with salt-bridges to R65 mediated by the CDR3α loop **(A,B)**. TCR represented by the 1BD2 file (see Figure 1B) utilizes a concave *mortise*, wherein R65 also forms salt bridges. 2VLR’s CDR3α contacts Q155 in a different strategy (see text).

Protein data bank files available for TCR:pHLA-A2 solved crystallographic structures (as listed in Figure 1A) were used to obtain the most likely nucleotide codons of the TCRA chain by reverse translation using the algorithms available at the *SMS*.^1^ Identification of Vα and Jα usage (*IMGT/V-Quest*) and junctional analysis (*IMGT/JunctionAnalysis*) among these TCR were performed by the *IMGT* algorithms^2^ and the results are shown in Figure 1B. Notice, all the CDR3α joints use extensive *N-nucleotide additions* (a hallmark of TCRVA somatic DNA rearrangements) to create a diverse set of amino acid sequences used within the solved structures. With 54 Vα and 61 Jα, TCRVA is unique among antigen receptors, and continuous rearrangement at TCRA ensures pHLA selects TCR (1). Here, we have undertaken a comprehensive analysis of each of the TCR:pHLA-A2 structures to examine the contacts made between each CDR3α loop and pHLA-A2 after we noticed that *alpha-dominant*, *alpha/beta*, and *beta-dominant* TCR binding all involved CDR3α contact with the MHC. Shown in Figures 2A–F is this conspicuous contact that all CDR3α make with the α-1 helix of HLA-A2. Note that all CDR3α make closest contact at the *same motif* centered on amino acid (a.a.) R65; 2VLR is the exception (Figures 2E,F).

### R65-joint

As shown in Figure 2 (compiled in Figure 1B), individual CDR3α rearrangements lead to structurally distinct types of contact with the *R65 motif*, principally, projection-type (*dovetail*), concave-type (*mortise*), or flat-type (*dado*), all best appreciated with space-filled models. For example, the *dovetail* joint of the A6 TCR (in 1AO7, 3PWP, and 3H9S complexes) fits W101 into the complimentary *slot* made by the side-chains of the *R65 motif*, i.e., within the α-helical secondary structure of the α-1 helix (Figures 2A,B). W101 is located on the lateral side of the CDR3α loop (i.e., the arm of the parabolic loop that faces away from the groove), and close contacts with α-1 are mediated by the arm of CDR3α that faces into the groove, i.e., ~3 Å contacts involving salt bridges (R65NE:D99OD1; R65NH2:T98OG1). Interestingly, the closest contact with the peptide also involves D99, i.e., Y5OH:D99N (peptide contacts listed in Figure 1B).

### Mortise

Looking further into the *R65-motif* connections demonstrates the use of a *mortise*, i.e., a CDR3α *lock* for the R65 *key*. As illustrated in Figures 2C,D, this is the most common type of contact and involves salt bridge formation between an acidic group(s) in CDR3α and one or more N of R65. One such joint involves N-H-N contact (*dado*-type of 304L); also, one of the contacts is shifted to Q72 by the 3GSN TCR (Figure 1B), and the 2VLR TCR is in less contact with α-1 helix (i.e., ~5 Å to R65); however, contacts the α-2 helix *via* a strikingly congruent *mortise* involving Q155 (Figures 2E,F). Indeed, 2VLR’s CDR3α seems like an alternative solution among these structures.

### CDR2α/α-2 helix interface

**Figure 3:**
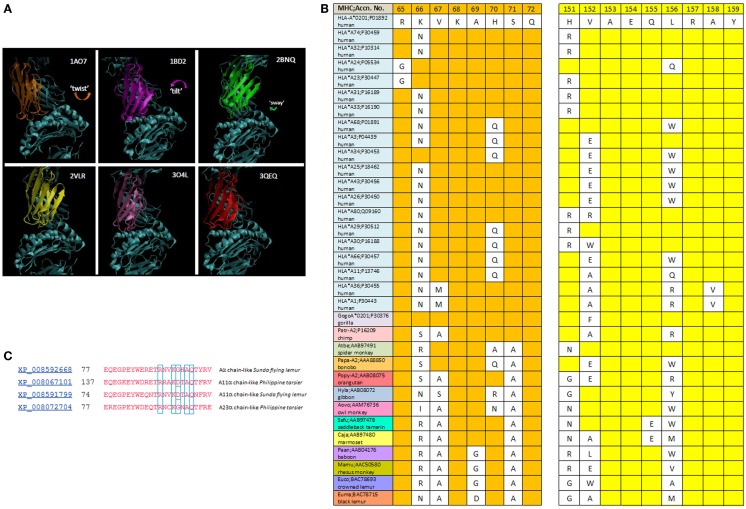
**(A)** The *twist/tilt/sway* of TCR-Vα relative to pHLA-A2; PDB files are denoted for each structure. TCR display a diversity of rotation *in-plane* to the groove {twist} with or without rotation perpendicular to the groove {tilting}; and this includes parallel (side-to-side) variation {sway}. Vα of each different TCR are colored; Vβ and pHLA-A2 are in cyan. **(B)** Alignment of the *R65 motif* and CDR2α-contact region among HLA-A alleles and non-human primate MHC *A-like* proteins. Sequences are from *NCBI* (*blink* analysis) with the HLA-A*0201 sequence as query (www.ncbi.nlm.nih.gov). **(C)** *NCBI* (*blast*) of HLA-A2 against *prosimians* taxid; alignment of different alleles (see text).

**Figure 4:**
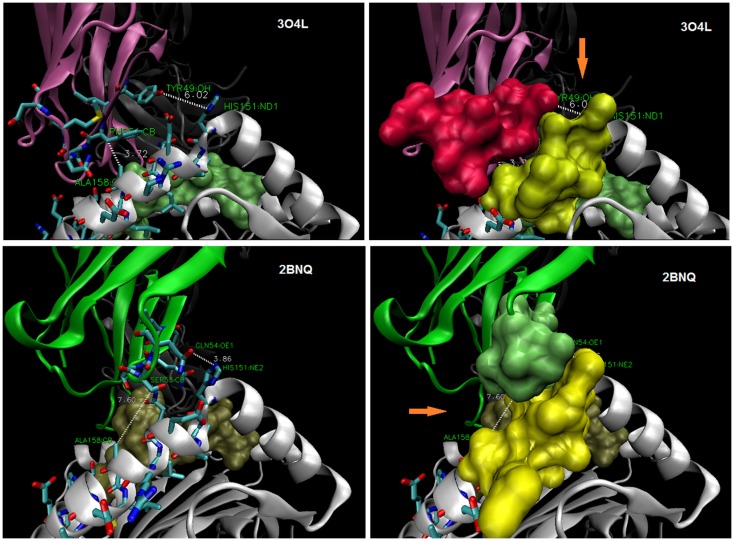
**TCR display interfaces with ~4 Å contact at either A158 (*top*) or at H151 (*bottom*)**. Thus, a steric site opposite (orange arrow) is available for occupancy by a larger side-chain (~6–7 Å). HLA-A30 (R151) bearing a permissive peptide would be expected to dock with 3O4L-like TCR, but not 2BNQ-like TCR; HLA-A1 (V158) with 2BNQ-like, but not 3O4L-like TCR. CDR2α and α2-helix (a.a. 151–158) side chains as stick (left), and space-filled (right); peptides are shown as space-filled.

The *R65-joint* is consistent with a range of TCR *twist/tilt/sway* (rotations about the plane of the pHLA *top face*) such that ~4 Å juxtaposition of CDR2α over HLA a.a. 151–158 is achieved (Figure 3A). Alignment of distant HLA-A alleles with A*0201 (Figure 3B) reveals that H151 of A2 is R151 in A-74, A-31, A-33, A-29, A-30, A-32, A-23, and A-80. Also, polymorphic is A158 of A2, which is V158 in A-36 and A-1. Other a.a. 151–158 α-2 polymorphisms are not oriented toward the TCR due to the α-helix. While they might influence allogeneic peptide identity, and thus indirectly the *R65-joint* (see below), A158V and H151R clearly *define* the interface. Since closer contacts would be expected for those CDR2α contacting A158 when the two –CH_3_ groups replace two –H on the pos. 158 a.a. Cβ, i.e., V158 (as found in HLA alleles, A-1 and A-36), and too, H151R could decrease contact distances (a longer side chain), it follows that all of these TCR maintain the *R65-joint* and the marginal contact with the α-2 helix by some shared mechanism. Moreover, it leads to an apparent steric consideration with respect to which allotypes are recognized by a given TCR (see Figure 4).

### Conservation

R65-X-X-K-A/G-X-S/A-Q72 is conserved in nearly all *primate* MHC *A-like* molecules (*black lemurs* are exceptions, with an A69D disruption; Figure 3B). Interestingly, *baboon*, *rhesus*, and *crowned lemur* have an A69G substitution, but this would substantively conserve motif structure. HLA-A24, -23 (as shown) do not have R65, but interestingly, variants of both do, e.g., A*2424, and A*2429. PDB 3W0W (TCR:HIV-1, Nef peptide:A-2402) has a *mortise* involving CDR3α Q94-G-G-K97 contact with E62 of the α-1 helix. This shifts the *across-the-groove* joint, but the CDR2α/α-2 interface range is maintained (see Figure 6) in 3W0W, the TCR is more *twisted* than in any of the HLA-A2 complexes (see below). More interesting (Figure 3C) is the apparent disruption of the *R65 motif* in alleles of *Tarsius syrichta* and the *colugo*, *Galeopterus variegatus*, as this puts the motif in a common ancestor (11), some 79.6 Mya (*Cretaceous*), i.e., well before *Paleocene-Eocene*, when *lemuriform*s and *tarsiiform*s are thought to have diverged (17, 18).

### Role of the peptide in the R65-joint

**Figure 5:**
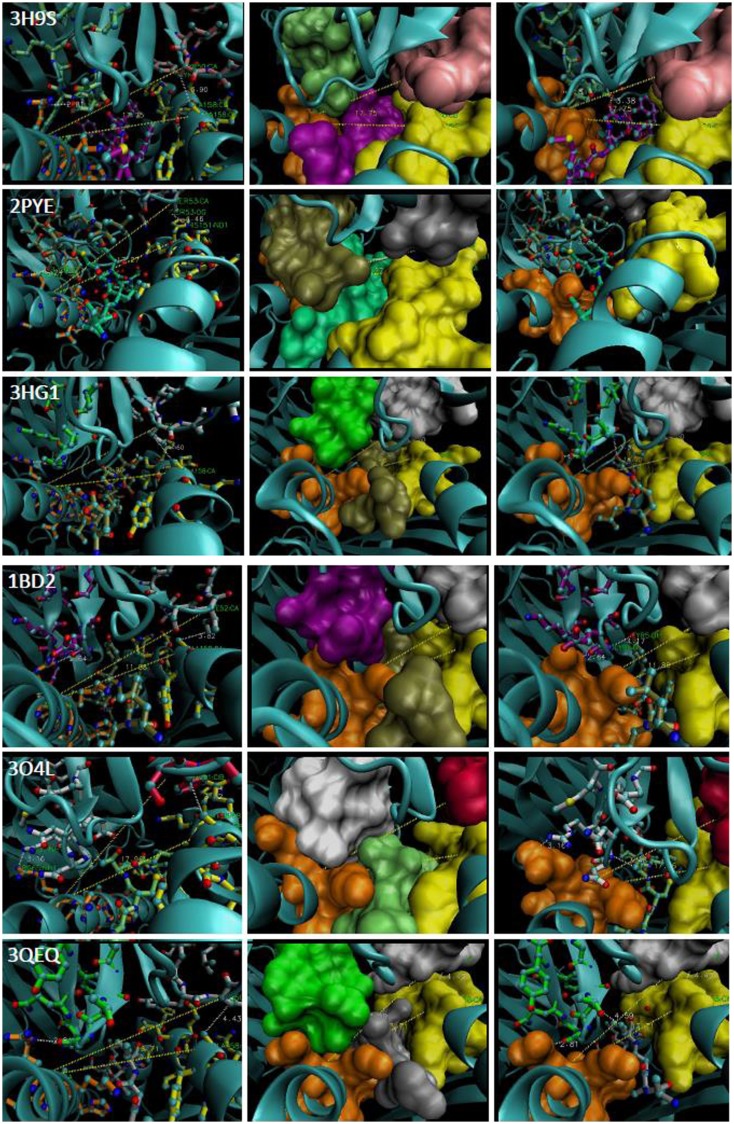
**The *CDR2α:R65:α-2 angle* of representative TCR:pHLA-A2**. PDB file is denoted in *far left* panels and *middle* and *right* panels reflect different views of each complex. The *R65 motif is* in orange and the H151-A158 region is in yellow. Contacting CDR3α a.a., lime (3H9S), tan (2PYE), green (3HG1), magenta (1BD2), white (3O4L), light green (3QEQ). CDR2α: pink (3H9S), silver (2PYE), white (3HG1), white (1BD2), rose (3O4L), and white (3QEQ); cyan ribbon alpha carbon backbones. Peptides: magenta (3H9S), foam (2PYE), tan (3HG1), tan (1BD2), lime (3O4L), and silver (3QEQ). The *R65-angle* was measured with *VMD* (shown as yellow trace). CDR3α:peptide contacts are shown as white trace.

**Figure 6:**
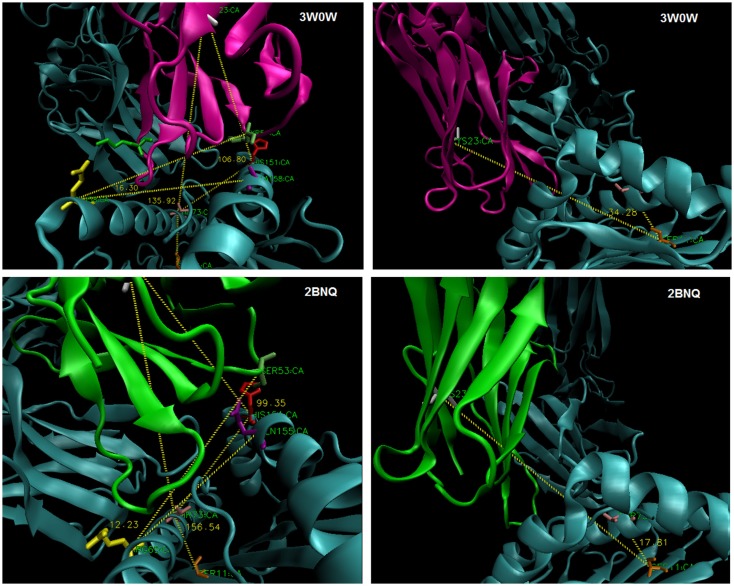
**Estimating TCR *twist/tilt/sway:* (left) measuring an angle across the groove to C22/3/4 of TCR-Vα (“twist”) and perpendicular to the groove to C22/3/4 (“tilt”); (right) measuring an angle parallel to the groove (“sway”)**.

As shown in Figure 5, the peptide contacts CDR3α in a fashion compatible with the *angle* between the *R65-joint* and the polymorphic contacts with MHC, *viz*., the CDR2α/α-2 helix interface. Within the structures examined here is displayed a consistent peptide interaction with what could be described as the arm of the CDR3α loop that faces away from R65. The closest contact of this nature among the examined complexes is in 3HG1, which is interesting because this peptide assumes an extended (*less bulged*) structure, and the angle between the CDR2α contact residue (alpha carbon), the R65 alpha carbon, and the α2-helix contact residue (alpha carbon) (*viz*., the *CDR2α:R65:α-2 angle*) is the largest amongst the structures at 18.90°(Figure 5). Interestingly, there is no direct correlation between this angle and the *closeness* of peptide contact (i.e., when we compare all the structures). However, the *CDR2α:R65:α-2 angle* does correlate with the overall orientation of the TCR on pHLA-A. For example, 2BNQ with a “flat” angle at 12.23° is tilted similarly to 3O4L, but is more twisted than 3O4L (Figure 3A); thus, the lack of “twist” for 3O4L correlates with its increased *R65-angle*, 17.96°, as would be the expected geometry. However, 3QEQ and 3W0W have about the same “tilt,” but 3W0W is quite more twisted; here, more “twist” correlated with an increased *R65-angle*. Therefore, *twisting* (ω) of the TCR in the plane of the groove seems dependent on the side-to-side *sway* (∂) parallel to the groove in its exact relationship to *tilting* (λ), i.e., toward the α-1 helix, at least with respect to increasing or decreasing the *R65-angle*, or *pitch* (φ). A plausible formula for the mechanism, based upon our estimates of these parameters, is the following (see Figure 6 and Table 1, for compiled data).

kφ=[∂÷(λ+∂)](ω)

**Table 1:** **Predicting the *R65-angle* from the orientation of TCR-Vα on pHLA-A**.

PDB	ω°	λ°	∂°	$\varphi_{\text{measured}}^{{}^{\circ}}$	k*	$\varphi_{\text{calculated}}^{{}^{\circ}}$
1AO7	96.12	128.94	39.48	17.93	1.26	22.53
3HG1	91.51	132.66	36.24	18.90	1.04	19.63
3O4L	89.00	141.85	29.02	17.96	0.84	15.11
2BNQ	99.35	156.54	17.81	12.23	0.83	10.15
3QEQ	93.32	135.26	34.26	13.71	1.38	18.86
3GSN	87.75	145.76	25.42	17.92	0.73	13.03
3W0W	106.80	135.92	34.28	16.30	1.32	21.51
3UTT	95.79	140.47	30.70	24.28	0.71	17.18
4QOK	103.34	131.06	37.67	19.73	1.17	23.07
4JFD	97.84	131.87	36.89	21.14	1.01	21.34
4EUP	97.32	140.30	30.35	16.80	1.03	17.31

*Estimated *twist*/*tilt/sway* of the TCR (from Vα) relative to the *R65-angle* and calculation*.

**Figure 7:**
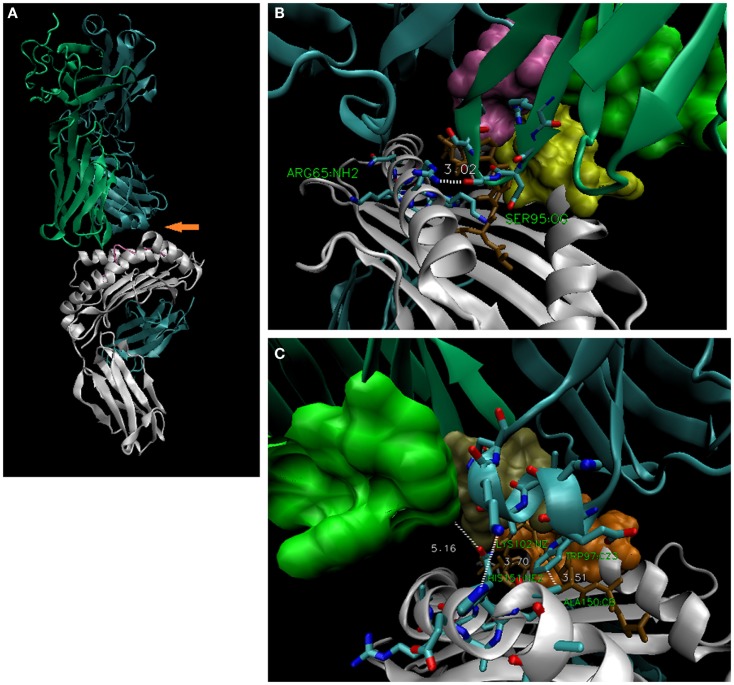
**The 3UTT TCR contacts R65 via CDR3α, but contacts the α-2 helix via CDR3β**. **(A)** Overall structure of 3UTT; orange arrow indicates *unusual* (here) across-the-groove contact between CDR3β and H151 [3.70 Å; bottom right, **(C)**]. Note CDR3α makes “usual” R65-joint [3.02 Å; upper right, **(B)**]. Like 2VLR, this *seems* an alternate strategy to the same distance of TCR contacts with the polymorphic a.a. 151–158 subregion. In this case, the interface was directly selected *via* the rearranged CDR3β; in all others, it is indirect *via* the described geometry; actual frequency for these TCR strategies is not known.

*k indicates deviation between values for φ. Mean *k* = 1.03 ± 0.23 (*s*), *n* = 11; *t* = 0.43, μ_o_ = 1.00; *p* = 0.67; thus (overall) φ values are not statistically different; 99% CI, *k* = 1.25–0.81; two-tailed Student’s *t-*test calculator tool @ http://in-silico.net/tools/statistics/ttest. For 3UTT, the closest contact with α-2 helix is *via* CDR3β (K102; Figure 7), which was used to measure the *R65-angle*.

*“Twist” (ω): measuring the angle: T73 (α-1 helix):H151 (α-2 helix):C22/3/4 (TCR-Vα)*.

*“Tilt” (λ): measuring the angle: S11 (β-1 stand):T73 (α-1 helix):C22/3/4 (TCR-Vα)*.

*“Sway” (∂): measuring the angle: T73 (α-1 helix):S11 (β-1 strand):C22/3/4 (TCR-Vα)*.

*“Pitch” (φ): *R65-angle* (CDR2α contact a.a.:R65:α-2 helix contact a.a.), related by: kφ = [∂ ÷ (λ + ∂)](ω)*.

Angles and contacts for PDB files not previously shown: 1AO7: Y50:R65:A158@17.93°, 2BNQ:S53:R65:Q155@12.23°, 4QOK: Y51:R65:A158@19.73°, 3GSN: I52:Q72:A158@17.92°, 4JFD: Y51:R65:A158@21.14°, 3UTT:K102β:R65:H151@24.28°, and 4EUP: Y52:R65:A158@16.80°.

One testable (19–21) idea is that peptide contacts stabilize dynamics and the CDR2α/α-2 helix interface. Perhaps, a “transition state,” involving key TCR interactions with the MHC, exists initially, followed by peptide interactions with the TCR being “scanned” in a two-step mechanism (22, 23). Alternatively, the TCR may “scan-clamp,” where peptide interactions come first (24–26), or peptide and MHC contacts might occur at the same time (14). Importantly, the *R65-joint* mechanism is not incompatible with any of these ideas; indeed, different rearrangements might utilize different dynamics to get to the same structural geometry.

The corollary that the *R65-angle* of these obviously *selected* TCR reflects deleted (*not-selected*) thymocytes yielding *closer* or *more distant* CDR2α/α-2 helix contacts is intriguing. In other words, a mature T-cell alloreactive capacity is selected-for *via* CDR3α that can do the *R65-joint*. Clearly, exceptions are 2VLR (as discussed), and notably 3UTT, wherein CDR3β assumes the ~4 Å contact with the α-2 helix, at H151. In this structure, the closest CDR3α contact is ~5 Å from Q155 (Figure 7). Thus, in the case of 3UTT, the interface of the TCR with a.a. 151–158 polymorphic positions appears to have been directly selected-for, i.e., the other TCR utilizes the indirect *across-the-groove* Vα geometry described herein.

## Conclusion

The idea that CDR2 and/or CDR1 have “co-evolved” with the MHC with the product being conserved/predictable contacts between them (4) has been disputed (27–29). For instance, co-receptors have been suggested as the true selective agents (28), and TCR have been selected in MHC knock-out mice independently of MHC (29). Nevertheless, CDR1/2 and MHC *are* clearly germ-line encoded, and any observations of conservative interactions across phylogeny are indeed evidence for “co-evolution” *per se*; what particular mechanism of thymic selection dictates it is still debatable. However, it must be considered that the somatic mechanism of CDR3 has had to entail with MHC polymorphism for some 400 My (30); and indeed, that the TCR repertoire is inherently alloreactive (1, 11).

The analysis presented here suggests a novel structural mechanism for MHC control of TCR diversity, and may help explain the enigmatic biology of T-cell alloreactivity. Thus, *somatic* CDR3α appears *selected* for TCR contact with *allo*-HLA-A by virtue of the *R65-joint* geometry explained herein, manifest in the TCR repertoire as the germline CDR2α/α-2 helix interface. Seemingly *unusual* 3UTT, wherein the interface is apparently directly selected-for *via* CDR3β, still utilized the R65-joint (S95O:R65NH2, 3.02 Å), and crucially maintained ~4 Å contact at the same α-2 helix position (βK102N2:H151NE2, 3.70 Å). Indeed, in both the 2VLR and 3UTT structures, TCR strategies for maintaining contact with the α-2 helix polymorphic positions seem like *exceptions to the rule*. Although, to be clear, the actual relative frequency of these different strategies within the TCR repertoire is not known. Nevertheless, the consistent use of the *R65-joint* geometry, even among these available structures, certainly hints at a rather *straightforward* hypothesis. Thus, TCR with CDR3α’s yielding TCR:pHLA-A2 complexes with the CDR2α/α-2 helix interface below or above ~4 Å (exception being 3UTT-like TCR) are proposed to be *theoretically* not selected. That surviving thymocytes *turn out to be* the *best* TCR bearers for protective immunity is *assumed* (this seems essential); what is clear, is that part of the immune system does respond directly against *allo*-HLA class I molecules for a biologically apparent reason. Indeed, *R65-joint* bioinformatics (as indicated) are consistent with the *emergence* of HLA-C and KIR genes (10). Maternal *u*NK cells induce fetal trophoblast-mediated re-modeling of the maternal circulation; yet, HLA-C:KIR is restricted to the *higher* primates (31). The structural *R65 motif* in a shared *prosimian* ancestor (11, 17, 18), that KIR and TCR bind to overlapping sites on pHLA-A molecules (10); pseudogenes and orphan receptors in extant human KIR genes (10, 31); and the balance of inflammatory/non-inflammatory cytokines (32), all tempt speculation that the *R65-joint* had/has a role in pregnancy. Finally, while several elegant mechanisms have been described for maintaining maternal tolerance against the fetal paternal allotype (31, 32); the *R65-joint* might facilitate fetal CD8 T cells to “reject” infiltrating maternal cells *via* the unshared HLA-A allele, perhaps in the second or third trimesters (33). Obviously, as gestation became more prolonged in primates, alleles containing the motif could have been favored.

## Conflict of Interest Statement

The author declares that he has no conflicts of interest regarding this research, manuscript, or its publication; and is solely responsible for the research and the writing of the manuscript.

## Footnotes

^1^http://www.bioinformatics.org

^2^http://www.imgt.org
